# Di-μ-chlorido-bis­(chlorido{2,2′-[3-(1*H*-imidazol-4-ylmeth­yl)-3-aza­pentane-1,5-di­yl]diphthalimide}copper(II))

**DOI:** 10.1107/S1600536809045565

**Published:** 2009-11-04

**Authors:** Zhao-Peng Qi, Ai-Dong Wang, Hui Zhang, Xi-Xi Wang

**Affiliations:** a Department of Chemistry, Huangshan University, Huangshan 245041, People’s Republic of China

## Abstract

The centrosymmetric dinuclear Cu^II^ complex, [Cu_2_Cl_4_(C_24_H_21_N_5_O_4_)_2_], was synthesized by the reaction of CuCl_2_·2H_2_O with the tripodal ligand 2,2′-[3-(1*H*-imid­azol-4-ylmeth­yl)-3-aza­pentane-1,5-di­yl]diphthalimide (*L*). Each of the Cu^II^ ions is coordinated by two N atoms from the ligand, two bridging Cl atoms and one terminal Cl atom. The Cu^II^ coordination can be best be described as a transition state between four- and five-coordination, since one of the bridging Cl atoms has a much longer Cu—Cl bond distance [2.7069 (13) Å] than the other [2.2630 (12) Å]. In addition, the Cu⋯Cu distance is 3.622 (1) Å. The three-dimensional structrure is generated by N—H⋯O, C—H⋯O and C—H⋯Cl hydrogen bonds and π–π inter­actions [centroid–centroid distances = 3.658 (4) and 4.020 (4) Å].

## Related literature

For the synthesis, see: Qi *et al.* (2008 ▶). For the use of imidazole-containing tripodal ligands in supra­molecular chemistry and new functional materials, see: Higa *et al.* (2007 ▶); Kong *et al.* (2005 ▶); Katsuki *et al.* (2002 ▶). For a related structure with a similar coordination geometry around the metal atom, see: Yu *et al.* (2009 ▶).
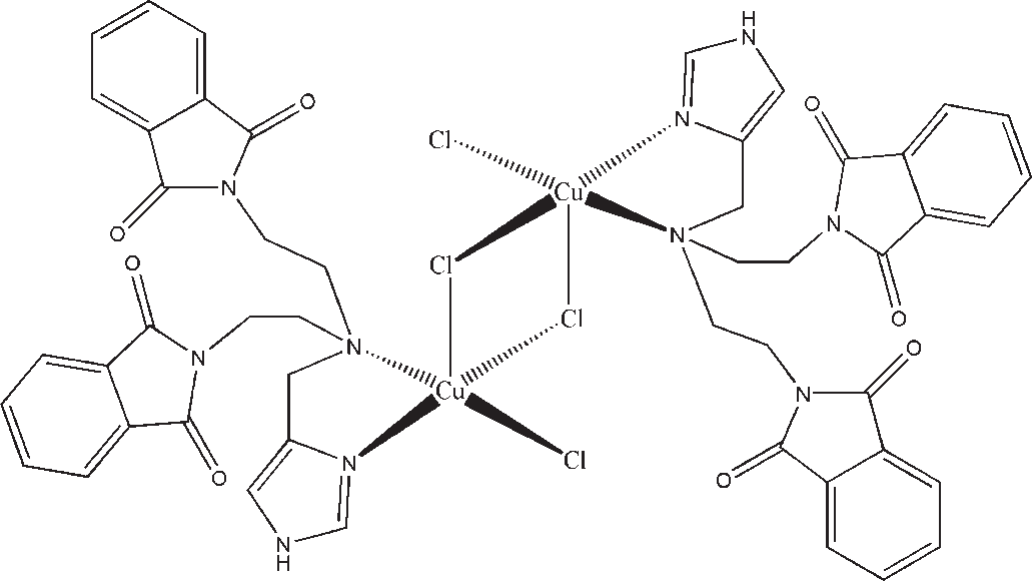


## Experimental

### 

#### Crystal data

[Cu_2_Cl_4_(C_24_H_21_N_5_O_4_)_2_]
*M* *_r_* = 1155.80
Monoclinic, 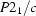

*a* = 8.4351 (9) Å
*b* = 14.6867 (16) Å
*c* = 20.1448 (19) Å
β = 105.593 (4)°
*V* = 2403.8 (4) Å^3^
*Z* = 2
Mo *K*α radiation
μ = 1.17 mm^−1^
*T* = 293 K
0.2 × 0.1 × 0.1 mm

#### Data collection

Bruker SMART CCD area-detector diffractometer
Absorption correction: multi-scan (*SADABS*; Bruker, 2001 ▶) *T*_min_ = 0.86, *T*_max_ = 0.89
11745 measured reflections
4218 independent reflections
3394 reflections with *I* > 2σ(*I*)
*R*_int_ = 0.047

#### Refinement

*R*[*F* ^2^ > 2σ(*F* ^2^)] = 0.069
*wR*(*F* ^2^) = 0.150
*S* = 1.17
4218 reflections
325 parameters
H-atom parameters constrained
Δρ_max_ = 0.61 e Å^−3^
Δρ_min_ = −0.30 e Å^−3^

### 

Data collection: *SMART* (Bruker, 2001 ▶); cell refinement: *SAINT* (Bruker, 2001 ▶); data reduction: *SAINT*; program(s) used to solve structure: *SHELXS97* (Sheldrick, 2008 ▶); program(s) used to refine structure: *SHELXL97* (Sheldrick, 2008 ▶); molecular graphics: *SHELXTL* (Sheldrick, 2008 ▶); software used to prepare material for publication: *SHELXTL*.

## Supplementary Material

Crystal structure: contains datablocks I, global. DOI: 10.1107/S1600536809045565/kp2236sup1.cif

Structure factors: contains datablocks I. DOI: 10.1107/S1600536809045565/kp2236Isup2.hkl

Additional supplementary materials:  crystallographic information; 3D view; checkCIF report

## Figures and Tables

**Table 1:** Selected bond lengths (Å)

Cu1—N2	1.932 (4)
Cu1—N1	2.211 (4)
Cu1—Cl1	2.2431 (15)
Cu1—Cl2^i^	2.2630 (12)
Cu1—Cl2	2.7069 (13)

Symmetry code: (i) 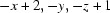.

**Table 2:** Hydrogen-bond geometry (Å, °)

*D*—H⋯*A*	*D*—H	H⋯*A*	*D*⋯*A*	*D*—H⋯*A*
N3—H3*A*⋯O3^ii^	0.86	2.37	3.022 (6)	133
C3—H3*B*⋯Cl1^iii^	0.93	2.65	3.445 (6)	144
C4—H4*A*⋯O2^ii^	0.93	2.45	3.131 (7)	131
C6—H6*B*⋯O2	0.97	2.51	2.870 (7)	102
C15—H15*A*⋯Cl1^i^	0.97	2.82	3.769 (5)	165
C20—H20*A*⋯O1^iv^	0.93	2.53	3.218 (9)	131

Symmetry codes: (i) 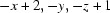; (ii) 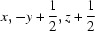; (iii) 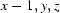; (iv) 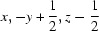.

**Fig. 1.**
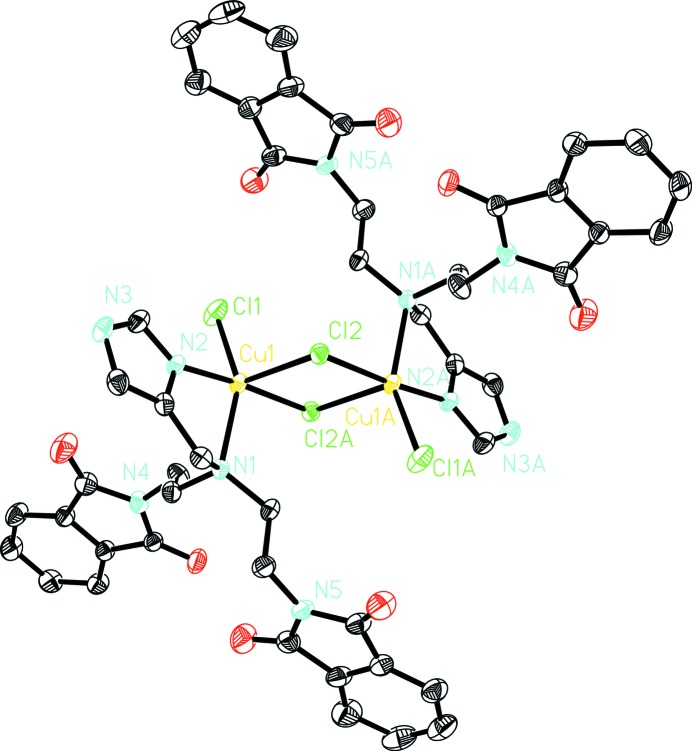
The molecular structure of (I) with atom labelling and 30% probability displacement ellipsoids for non-H atoms.

**Fig. 2.**
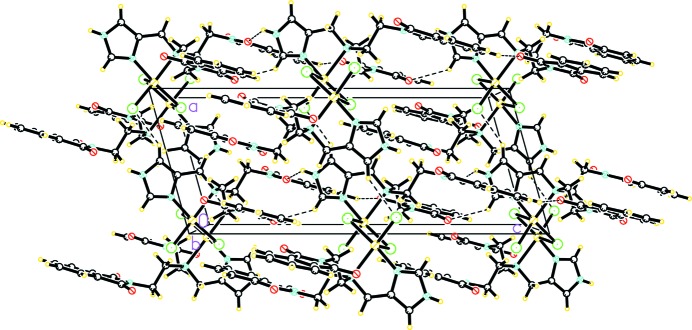
The three-dimensional packing of (I) viewed down the b axisrealized by N—H···O, C—H···O, C—H···Cl hydrogen-bonds (dashed lines) and π–π interactions.

## supplementary crystallographic information

### Comment

In recent years, imidazole-containing tripodal ligands have attracted much attention for their extensive use in supramolecular chemistry and new functional materials (Higa, *et al.*, 2007; Kong *et al.*, 2005; Katsuki,*et al.*, 2002). Here, we synthesized a new tripodal ligand *L*, 3-(imidazole-4-yl-methyl)-1,5-diphthalimido-3-azapentane, and reported its Cu^II^ complex.

In this complex, the Cu^II^ ion is coordinated by two N atoms from the ligand, two bridging Cl atoms and one terminal Cl atom. The two bridging Cl atoms are quite different, the equatorial Cl atom exbits a Cu—Cl distance of 2.2630 (12) Å, while that of the axial Cl atom is much longer with 2.7069 (13) Å (Table 1). Thus, the Cu^II^ coordination can be better described as a transition state between 4 and 5 coordination. In addition, the Cu—Cu distance is about 3.622 Å. A dimer of two monomeric units bridged by two chlorido ions reveals an inversion centre in the middle of the molecule (Fig. 1). The dimers are further connected to form the three-dimensional packing by N—H···O, C—H···O, C—H···Cl hydrogen-bonds and π–π interactions involving neighbouring phthalamide rings [Cg1···Cg2(-x+2,y+1/2,-z+1/2) = 3.658 (4) and Cg1···Cg3(-x+1,y+1/2,-z+1/2 = 4.020 (4) Å where Cg1, Cg2 and Cg2 are the centroids of the N5/C17/C18/C23/C24, C18–C23 and N4/C7/C8/C13/C14 rings, respectively (Fig. 2)].

### Experimental

The tripodal ligand *L*, 3-(imidazole-4-yl-methyl)-1,5-diphthalimido-3-azapentane, was synthesized by a literature method (Qi *et al.*, 2008). The title complex was synthesized as follows: a methanol solution (3 ml) of *L* (36.3 mg, 0.1 mmol) was added to a CH_3_CN solution (2 ml) of CuCl_2_.2H_2_O (17.0 mg, 0.1 mmol). Green crystals were obtained by slow evaporation of the solution in air for several days.

### Refinement

All H atoms were refined using a riding model. C—H values were set to 0.93 to 0.97 Å with *U*_iso_(H) = 1.2 *U*_eq_(C), and N—H values were set to 0.86 Å with *U*_iso_(H) = 1.2 *U*_eq_(N).

### Crystal data

**Table:**

[Cu_2_Cl_4_(C_24_H_21_N_5_O_4_)_2_]	*F*(000) = 1180
*M**_r_* = 1155.80	*D*_x_ = 1.597 Mg m^−^^3^
Monoclinic, *P*2_1_/*c*	Mo *K*α radiation, λ = 0.71073 Å
Hall symbol: -P 2ybc	Cell parameters from 1643 reflections
*a* = 8.4351 (9) Å	θ = 2.5–21.3°
*b* = 14.6867 (16) Å	µ = 1.17 mm^−^^1^
*c* = 20.1448 (19) Å	*T* = 293 K
β = 105.593 (4)°	Block, green
*V* = 2403.8 (4) Å^3^	0.2 × 0.1 × 0.1 mm
*Z* = 2	

### Data collection

**Table:**

Bruker SMART CCD area-detector diffractometer	4218 independent reflections
Radiation source: fine-focus sealed tube	3394 reflections with *I* > 2σ(*I*)
graphite	*R*_int_ = 0.047
φ and ω scans	θ_max_ = 25.0°, θ_min_ = 2.1°
Absorption correction: multi-scan (*SADABS*; Bruker, 2001)	*h* = −10→10
*T*_min_ = 0.86, *T*_max_ = 0.89	*k* = −17→9
11745 measured reflections	*l* = −22→23

### Refinement

**Table:**

Refinement on *F*^2^	Primary atom site location: structure-invariant direct methods
Least-squares matrix: full	Secondary atom site location: difference Fourier map
*R*[*F*^2^ > 2σ(*F*^2^)] = 0.069	Hydrogen site location: inferred from neighbouring sites
*wR*(*F*^2^) = 0.150	H-atom parameters constrained
*S* = 1.17	*w* = 1/[σ^2^(*F*_o_^2^) + (0.0656*P*)^2^ + 0.075*P*] where *P* = (*F*_o_^2^ + 2*F*_c_^2^)/3
4218 reflections	(Δ/σ)_max_ = 0.001
325 parameters	Δρ_max_ = 0.61 e Å^−^^3^
0 restraints	Δρ_min_ = −0.30 e Å^−^^3^
0 constraints	

### Special details

**Table:**

Geometry. All e.s.d.'s (except the e.s.d. in the dihedral angle between two l.s. planes) are estimated using the full covariance matrix. The cell e.s.d.'s are taken into account individually in the estimation of e.s.d.'s in distances, angles and torsion angles; correlations between e.s.d.'s in cell parameters are only used when they are defined by crystal symmetry. An approximate (isotropic) treatment of cell e.s.d.'s is used for estimating e.s.d.'s involving l.s. planes.
Refinement. Refinement of *F*^2^ against ALL reflections. The weighted *R*-factor *wR* and goodness of fit *S* are based on *F*^2^, conventional *R*-factors *R* are based on *F*, with *F* set to zero for negative *F*^2^. The threshold expression of *F*^2^ > σ(*F*^2^) is used only for calculating *R*-factors(gt) *etc*. and is not relevant to the choice of reflections for refinement. *R*-factors based on *F*^2^ are statistically about twice as large as those based on *F*, and *R*- factors based on ALL data will be even larger.

### Fractional atomic coordinates and isotropic or equivalent isotropic displacement parameters (Å^2^)

**Table:**

	*x*	*y*	*z*	*U*_iso_*/*U*_eq_	
Cu1	0.93018 (7)	0.11519 (4)	0.50753 (3)	0.0346 (2)	
Cl1	1.12292 (17)	0.19333 (11)	0.58499 (8)	0.0620 (4)	
Cl2	0.89142 (14)	−0.05739 (8)	0.54767 (6)	0.0369 (3)	
O1	0.7721 (7)	0.4237 (3)	0.4369 (2)	0.0851 (15)	
O2	0.9233 (5)	0.2868 (3)	0.26250 (19)	0.0620 (11)	
O3	0.6332 (6)	0.1249 (3)	0.1982 (2)	0.0799 (14)	
O4	0.5644 (5)	−0.1647 (3)	0.2630 (2)	0.0696 (12)	
N1	0.7125 (5)	0.1113 (2)	0.41720 (19)	0.0331 (9)	
N2	0.7596 (5)	0.1576 (3)	0.54753 (19)	0.0371 (9)	
N3	0.5988 (6)	0.2086 (3)	0.6073 (2)	0.0573 (13)	
H3A	0.5670	0.2339	0.6400	0.069*	
N4	0.8396 (5)	0.3347 (3)	0.3553 (2)	0.0426 (10)	
N5	0.6000 (5)	−0.0116 (3)	0.2470 (2)	0.0445 (10)	
C1	0.5748 (6)	0.0876 (3)	0.4473 (2)	0.0372 (11)	
H1A	0.4704	0.1049	0.4158	0.045*	
H1B	0.5733	0.0225	0.4553	0.045*	
C2	0.6000 (6)	0.1372 (3)	0.5129 (3)	0.0374 (12)	
C3	0.4990 (7)	0.1690 (4)	0.5504 (3)	0.0496 (14)	
H3B	0.3849	0.1645	0.5392	0.060*	
C4	0.7537 (7)	0.2019 (4)	0.6041 (3)	0.0517 (14)	
H4A	0.8446	0.2250	0.6368	0.062*	
C5	0.6910 (6)	0.2056 (3)	0.3885 (3)	0.0395 (12)	
H5A	0.6333	0.2423	0.4146	0.047*	
H5B	0.6248	0.2037	0.3410	0.047*	
C6	0.8551 (6)	0.2485 (3)	0.3918 (3)	0.0489 (14)	
H6A	0.9144	0.2582	0.4396	0.059*	
H6B	0.9193	0.2069	0.3720	0.059*	
C7	0.8081 (7)	0.4161 (4)	0.3837 (3)	0.0510 (14)	
C8	0.8283 (6)	0.4883 (3)	0.3344 (2)	0.0424 (12)	
C9	0.8152 (8)	0.5819 (4)	0.3369 (3)	0.0586 (16)	
H9A	0.7858	0.6107	0.3730	0.070*	
C10	0.8480 (8)	0.6310 (4)	0.2832 (3)	0.0598 (16)	
H10A	0.8419	0.6942	0.2835	0.072*	
C11	0.8889 (7)	0.5882 (4)	0.2298 (3)	0.0557 (15)	
H11A	0.9083	0.6232	0.1943	0.067*	
C12	0.9023 (7)	0.4950 (4)	0.2270 (3)	0.0484 (13)	
H12A	0.9306	0.4664	0.1906	0.058*	
C13	0.8722 (6)	0.4459 (3)	0.2804 (3)	0.0410 (12)	
C14	0.8824 (6)	0.3467 (3)	0.2942 (3)	0.0412 (12)	
C15	0.7243 (6)	0.0443 (3)	0.3632 (2)	0.0383 (12)	
H15A	0.7659	−0.0126	0.3857	0.046*	
H15B	0.8046	0.0665	0.3405	0.046*	
C16	0.5645 (6)	0.0249 (4)	0.3085 (3)	0.0477 (13)	
H16A	0.4994	−0.0186	0.3261	0.057*	
H16B	0.5013	0.0806	0.2971	0.057*	
C17	0.6355 (7)	0.0435 (4)	0.1965 (3)	0.0555 (15)	
C18	0.6740 (7)	−0.0193 (4)	0.1452 (3)	0.0559 (15)	
C19	0.7175 (9)	−0.0007 (5)	0.0860 (3)	0.083 (2)	
H19A	0.7253	0.0587	0.0711	0.099*	
C20	0.7496 (10)	−0.0755 (7)	0.0492 (3)	0.089 (2)	
H20A	0.7801	−0.0659	0.0086	0.107*	
C21	0.7373 (8)	−0.1627 (6)	0.0710 (4)	0.075 (2)	
H21A	0.7602	−0.2112	0.0454	0.090*	
C22	0.6916 (7)	−0.1796 (5)	0.1305 (3)	0.0638 (17)	
H22A	0.6835	−0.2390	0.1453	0.077*	
C23	0.6583 (7)	−0.1073 (4)	0.1675 (3)	0.0506 (14)	
C24	0.6043 (7)	−0.1033 (4)	0.2314 (3)	0.0504 (14)	

### Atomic displacement parameters (Å^2^)

**Table:**

	*U*^11^	*U*^22^	*U*^33^	*U*^12^	*U*^13^	*U*^23^
Cu1	0.0370 (4)	0.0344 (4)	0.0361 (4)	0.0013 (3)	0.0159 (3)	−0.0045 (3)
Cl1	0.0480 (8)	0.0709 (11)	0.0690 (10)	−0.0078 (7)	0.0189 (7)	−0.0349 (8)
Cl2	0.0405 (7)	0.0386 (7)	0.0359 (7)	0.0054 (5)	0.0173 (5)	0.0011 (5)
O1	0.145 (5)	0.064 (3)	0.068 (3)	0.003 (3)	0.067 (3)	0.005 (2)
O2	0.099 (3)	0.039 (2)	0.054 (2)	0.013 (2)	0.031 (2)	−0.0029 (19)
O3	0.123 (4)	0.044 (3)	0.076 (3)	−0.005 (3)	0.031 (3)	0.005 (2)
O4	0.094 (3)	0.056 (3)	0.063 (3)	−0.016 (2)	0.029 (2)	0.002 (2)
N1	0.040 (2)	0.030 (2)	0.034 (2)	0.0029 (17)	0.0180 (18)	0.0043 (17)
N2	0.038 (2)	0.043 (2)	0.034 (2)	0.0052 (19)	0.0167 (19)	−0.0045 (19)
N3	0.067 (3)	0.064 (3)	0.050 (3)	0.016 (3)	0.031 (3)	−0.010 (2)
N4	0.055 (3)	0.028 (2)	0.048 (3)	−0.001 (2)	0.021 (2)	0.008 (2)
N5	0.059 (3)	0.039 (3)	0.034 (2)	−0.007 (2)	0.011 (2)	−0.004 (2)
C1	0.036 (3)	0.036 (3)	0.042 (3)	−0.007 (2)	0.014 (2)	0.005 (2)
C2	0.044 (3)	0.034 (3)	0.042 (3)	0.004 (2)	0.024 (2)	0.007 (2)
C3	0.049 (3)	0.052 (4)	0.055 (4)	0.010 (3)	0.025 (3)	0.007 (3)
C4	0.058 (4)	0.056 (4)	0.044 (3)	0.006 (3)	0.018 (3)	−0.011 (3)
C5	0.046 (3)	0.036 (3)	0.040 (3)	0.006 (2)	0.019 (2)	0.005 (2)
C6	0.052 (3)	0.036 (3)	0.058 (4)	0.000 (2)	0.014 (3)	0.016 (3)
C7	0.066 (4)	0.046 (3)	0.047 (3)	0.000 (3)	0.026 (3)	0.009 (3)
C8	0.056 (3)	0.034 (3)	0.041 (3)	0.000 (2)	0.019 (3)	0.002 (2)
C9	0.093 (5)	0.035 (3)	0.053 (4)	−0.007 (3)	0.028 (3)	−0.006 (3)
C10	0.088 (5)	0.027 (3)	0.066 (4)	−0.009 (3)	0.023 (4)	0.003 (3)
C11	0.074 (4)	0.045 (4)	0.053 (4)	−0.008 (3)	0.024 (3)	0.015 (3)
C12	0.068 (4)	0.042 (3)	0.041 (3)	−0.005 (3)	0.026 (3)	0.005 (3)
C13	0.053 (3)	0.028 (3)	0.043 (3)	−0.006 (2)	0.015 (2)	0.001 (2)
C14	0.056 (3)	0.032 (3)	0.036 (3)	0.000 (2)	0.013 (2)	0.001 (2)
C15	0.045 (3)	0.034 (3)	0.040 (3)	−0.001 (2)	0.018 (2)	−0.006 (2)
C16	0.047 (3)	0.053 (4)	0.043 (3)	0.000 (3)	0.012 (2)	−0.006 (3)
C17	0.067 (4)	0.046 (4)	0.048 (3)	−0.001 (3)	0.006 (3)	0.003 (3)
C18	0.066 (4)	0.062 (4)	0.038 (3)	−0.005 (3)	0.011 (3)	−0.004 (3)
C19	0.118 (6)	0.080 (5)	0.058 (4)	−0.005 (5)	0.037 (4)	0.010 (4)
C20	0.107 (6)	0.123 (7)	0.044 (4)	−0.010 (5)	0.033 (4)	−0.012 (5)
C21	0.066 (4)	0.097 (6)	0.066 (5)	−0.009 (4)	0.023 (4)	−0.031 (4)
C22	0.057 (4)	0.065 (4)	0.063 (4)	−0.007 (3)	0.005 (3)	−0.018 (3)
C23	0.051 (3)	0.049 (4)	0.047 (3)	−0.005 (3)	0.005 (3)	−0.008 (3)
C24	0.055 (3)	0.052 (4)	0.041 (3)	−0.009 (3)	0.008 (3)	−0.006 (3)

### Geometric parameters (Å, °)

**Table:**

Cu1—N2	1.932 (4)	C5—H5B	0.9700
Cu1—N1	2.211 (4)	C6—H6A	0.9700
Cu1—Cl1	2.2431 (15)	C6—H6B	0.9700
Cu1—Cl2^i^	2.2630 (12)	C7—C8	1.493 (7)
Cu1—Cl2	2.7069 (13)	C8—C9	1.382 (7)
Cl2—Cu1^i^	2.2630 (12)	C8—C13	1.386 (7)
O1—C7	1.196 (6)	C9—C10	1.389 (8)
O2—C14	1.192 (6)	C9—H9A	0.9300
O3—C17	1.198 (6)	C10—C11	1.368 (8)
O4—C24	1.202 (6)	C10—H10A	0.9300
N1—C1	1.489 (5)	C11—C12	1.376 (7)
N1—C15	1.489 (5)	C11—H11A	0.9300
N1—C5	1.492 (6)	C12—C13	1.375 (6)
N2—C4	1.325 (6)	C12—H12A	0.9300
N2—C2	1.373 (6)	C13—C14	1.481 (7)
N3—C4	1.329 (6)	C15—C16	1.522 (7)
N3—C3	1.358 (7)	C15—H15A	0.9700
N3—H3A	0.8600	C15—H15B	0.9700
N4—C7	1.382 (7)	C16—H16A	0.9700
N4—C14	1.383 (6)	C16—H16B	0.9700
N4—C6	1.452 (6)	C17—C18	1.485 (8)
N5—C24	1.385 (7)	C18—C19	1.366 (7)
N5—C17	1.392 (7)	C18—C23	1.387 (8)
N5—C16	1.453 (6)	C19—C20	1.393 (10)
C1—C2	1.472 (7)	C19—H19A	0.9300
C1—H1A	0.9700	C20—C21	1.367 (10)
C1—H1B	0.9700	C20—H20A	0.9300
C2—C3	1.364 (6)	C21—C22	1.377 (8)
C3—H3B	0.9300	C21—H21A	0.9300
C4—H4A	0.9300	C22—C23	1.368 (8)
C5—C6	1.506 (6)	C22—H22A	0.9300
C5—H5A	0.9700	C23—C24	1.479 (7)
			
N2—Cu1—N1	78.75 (15)	N4—C7—C8	105.7 (4)
N2—Cu1—Cl1	91.52 (13)	C9—C8—C13	121.1 (5)
N1—Cu1—Cl1	150.69 (11)	C9—C8—C7	131.1 (5)
N2—Cu1—Cl2^i^	173.87 (13)	C13—C8—C7	107.8 (5)
N1—Cu1—Cl2^i^	95.78 (10)	C8—C9—C10	116.9 (5)
Cl1—Cu1—Cl2^i^	94.61 (5)	C8—C9—H9A	121.5
N2—Cu1—Cl2	90.81 (12)	C10—C9—H9A	121.5
N1—Cu1—Cl2	94.67 (10)	C11—C10—C9	121.3 (5)
Cl1—Cu1—Cl2	113.23 (6)	C11—C10—H10A	119.3
Cl2^i^—Cu1—Cl2	86.86 (4)	C9—C10—H10A	119.3
Cu1^i^—Cl2—Cu1	93.14 (4)	C10—C11—C12	122.0 (5)
C1—N1—C15	110.8 (4)	C10—C11—H11A	119.0
C1—N1—C5	110.3 (3)	C12—C11—H11A	119.0
C15—N1—C5	110.8 (3)	C13—C12—C11	117.1 (5)
C1—N1—Cu1	103.7 (3)	C13—C12—H12A	121.5
C15—N1—Cu1	114.5 (3)	C11—C12—H12A	121.5
C5—N1—Cu1	106.4 (3)	C12—C13—C8	121.5 (5)
C4—N2—C2	106.6 (4)	C12—C13—C14	130.5 (5)
C4—N2—Cu1	136.2 (4)	C8—C13—C14	108.0 (4)
C2—N2—Cu1	117.1 (3)	O2—C14—N4	124.4 (5)
C4—N3—C3	108.8 (4)	O2—C14—C13	129.5 (5)
C4—N3—H3A	125.6	N4—C14—C13	106.1 (4)
C3—N3—H3A	125.6	N1—C15—C16	115.7 (4)
C7—N4—C14	112.5 (4)	N1—C15—H15A	108.4
C7—N4—C6	123.2 (4)	C16—C15—H15A	108.4
C14—N4—C6	123.5 (4)	N1—C15—H15B	108.4
C24—N5—C17	112.1 (4)	C16—C15—H15B	108.4
C24—N5—C16	125.1 (4)	H15A—C15—H15B	107.4
C17—N5—C16	122.8 (5)	N5—C16—C15	110.0 (4)
C2—C1—N1	108.0 (4)	N5—C16—H16A	109.7
C2—C1—H1A	110.1	C15—C16—H16A	109.7
N1—C1—H1A	110.1	N5—C16—H16B	109.7
C2—C1—H1B	110.1	C15—C16—H16B	109.7
N1—C1—H1B	110.1	H16A—C16—H16B	108.2
H1A—C1—H1B	108.4	O3—C17—N5	123.4 (6)
C3—C2—N2	108.4 (5)	O3—C17—C18	130.4 (6)
C3—C2—C1	134.8 (5)	N5—C17—C18	106.1 (5)
N2—C2—C1	116.8 (4)	C19—C18—C23	122.7 (6)
N3—C3—C2	106.1 (5)	C19—C18—C17	130.1 (6)
N3—C3—H3B	127.0	C23—C18—C17	107.2 (5)
C2—C3—H3B	127.0	C18—C19—C20	116.3 (7)
N2—C4—N3	110.1 (5)	C18—C19—H19A	121.8
N2—C4—H4A	125.0	C20—C19—H19A	121.8
N3—C4—H4A	125.0	C21—C20—C19	121.7 (6)
N1—C5—C6	110.9 (4)	C21—C20—H20A	119.2
N1—C5—H5A	109.5	C19—C20—H20A	119.2
C6—C5—H5A	109.5	C20—C21—C22	120.8 (7)
N1—C5—H5B	109.5	C20—C21—H21A	119.6
C6—C5—H5B	109.5	C22—C21—H21A	119.6
H5A—C5—H5B	108.0	C23—C22—C21	118.7 (7)
N4—C6—C5	112.7 (4)	C23—C22—H22A	120.7
N4—C6—H6A	109.0	C21—C22—H22A	120.7
C5—C6—H6A	109.0	C22—C23—C18	119.8 (5)
N4—C6—H6B	109.0	C22—C23—C24	131.4 (6)
C5—C6—H6B	109.0	C18—C23—C24	108.8 (5)
H6A—C6—H6B	107.8	O4—C24—N5	125.6 (5)
O1—C7—N4	125.1 (5)	O4—C24—C23	128.8 (5)
O1—C7—C8	129.2 (5)	N5—C24—C23	105.6 (5)

Symmetry codes: (i) −*x*+2, −*y*, −*z*+1.

### Hydrogen-bond geometry (Å, °)

**Table:**

*D*—H···*A*	*D*—H	H···*A*	*D*···*A*	*D*—H···*A*
N3—H3A···O3^ii^	0.86	2.37	3.022 (6)	133
C3—H3B···Cl1^iii^	0.93	2.65	3.445 (6)	144
C4—H4A···O2^ii^	0.93	2.45	3.131 (7)	131
C6—H6B···O2	0.97	2.51	2.870 (7)	102
C15—H15A···Cl1^i^	0.97	2.82	3.769 (5)	165
C20—H20A···O1^iv^	0.93	2.53	3.218 (9)	131

Symmetry codes: (ii) *x*, −*y*+1/2, *z*+1/2; (iii) *x*−1, *y*, *z*; (i) −*x*+2, −*y*, −*z*+1; (iv) *x*, −*y*+1/2, *z*−1/2.
